# Digital Breast Tomosynthesis Versus Additional Diagnostic Mammographic Views for the Evaluation of Asymmetric Mammographic Densities

**DOI:** 10.7759/cureus.9637

**Published:** 2020-08-10

**Authors:** Hira Waheed, Imrana Masroor, Shaista Afzal, Muhammad Ismail Alvi, Syed Jahanzeb

**Affiliations:** 1 Radiology, Aga Khan University Hospital, Karachi, PAK; 2 Orthopedics, Civil Hospital Karachi, Dow University of Health Sciences, Karachi, PAK

**Keywords:** additional view (av), digital breast tomosynthesis (dbt), ultrasound (u/s), radiographically dense breasts

## Abstract

Introduction

Many young females present with an advanced stage of breast cancer, which has a negative effect on the prognosis. Digital breast tomosynthesis is a new emerging imaging technique that aids in improving the specificity of mammography with subsequent early detection of breast cancer, especially in women with radiographically dense breasts. Tomosynthesis is subjectively preferred to conventional mammography and may offer superior diagnostic accuracy for the evaluation of breast lesions.

Method

Two breast radiologists retrospectively reviewed asymmetric densities using protocols that were institutional review board-approved in 185 patients aged 18 - 70 years (mean: 48 years) who underwent diagnostic mammography and tomosynthesis. Each asymmetric density was interpreted once with tomosynthesis and once with supplemental mammographic views; both modes included the mediolateral oblique and craniocaudal views in a fully crossed and balanced design by using a five-category Breast Imaging Reporting and Data System (BI-RADS) assessment and a probability-of-malignancy score. If the abnormality persisted and appeared benign or completely disappeared on both modalities, the agreement between additional views and tomosynthesis was determined by calculating Kappa value. If there was a discrepancy between additional views and tomosynthesis, the abnormality was subjected to ultrasound. In our study, 89 asymmetric densities were subjected to ultrasound.

Results

In a total of 182 cases, 84 (46.15%) were categorized as BIRADS-0; 97 (53.30 %) as BIRADS-I, and one (0.55 %) as BIRADS-II on an additional view. Among the asymmetric densities categorized as BIRADS-0 on additional mammography views, digital breast tomosynthesis categorized 72, six, five, and one patient as BIRADS-0, BIRADS-I, BIRADS-II, and BIRADS-IV, respectively. For densities categorized as BIRADS-I (97) on additional view, digital breast tomosynthesis categorized 10 and 87 densities as BIRADS-0 and BIRADS-I, respectively.

No change in the BIRADS category was observed among BIRADS-II and BIRADS-IV.

A significant difference was observed with the chi-square test among BIRADS categories assigned by an additional view and digital breast tomosynthesis with a p-value of < 0.001. There was, however, a substantial agreement among additional views and tomosynthesis with a kappa value of 0.767.

Conclusion

Our study results suggest that tomosynthesis may be equivalent to, if not more equivalent to, additional imaging in the assessment of mammographically-detected asymmetric densities, thus improving BI-RADS classification and patient management.

## Introduction

According to the World Health Organization (WHO), breast cancer is the most common cancer among women [1]. In Asia, Pakistan has the highest rate of breast cancer, ranking eighth in the world [2]. Many young women present at an advanced stage of breast cancer, which negatively affects prognosis [3].

Digital breast tomosynthesis (DBT) is an emerging imaging technique that creates cross-sectional images of the compressed breast. It aids in reducing false positives and provides equal or better sensitivity compared with mammography and improves the specificity of mammography, especially in women with radiographically dense breasts [4-5]. Literature has shown that women subjectively prefer tomosynthesis to conventional mammography, which may offer better diagnostic accuracy for the evaluation of breast lesions [6-8].

As DBT is an emerging new imaging technique, no work has been carried out in Pakistan on breast tomosynthesis to date. The goal of this study was to determine the agreement between additional views (AV) and DBT in the detection of asymmetric densities to assess the need for additional views in women undergoing DBT.

## Materials and methods

We conducted a retrospective study in the Department of Radiology, Aga Khan University Hospital, Karachi, Pakistan, from February 2018 to February 2019. A sample size of 187 women was calculated to yield a power of 80%, and confidence intervals (CI) of 95% using previously reported mean difference in the sensitivity of 89.7% vs. 78.9% for tomosynthesis vs. mammogram in the detection of malignancy [9]. Informed consent was waived.

The study included all adult women (18 to 70 years old) referred to the Radiology Department of Aga Khan University Hospital for diagnostic mammography with an additional one to two mammographic views (e.g., spot compression views), followed by single/two view breast tomosynthesis for parenchymal asymmetry.

Patients having microcalcification on mammography and those who had their prior imaging from outside our hospital were excluded.

We enrolled patients meeting the inclusion criteria in the study. Two consultant radiologists with ≥ 10 years of clinical experience in women’s imaging reviewed the diagnostic mammograms. They recorded findings on a standard proforma by the principal investigator. If any asymmetric density was found on a mammogram, it was analyzed on additional views and via DBT. If the abnormality persisted and appeared benign or completely disappeared on both modalities, the agreement between the two was that AV and DBT would be determined by calculating the Kappa value. In case of discrepancies between AV and DBT, the abnormality was subjected to ultrasound. All Breast Imaging Reporting and Data System (BIRADS) categories of mammograms, additional views, DBT, and ultrasound were recorded separately.

Standard mammographic views (craniocaudal and mediolateral oblique views of both breasts, along with additional views including cone compression and tomosynthesis) were performed on Siemens Mammomat Inspiration equipment (Siemens GmbH, Erlangen, Germany).

Tomosynthesis was performed using the Siemens Mammomat Inspiration with the wide-angle tomosynthesis option (Siemens GmbH, Erlangen, Germany). The dose was determined by an automatic exposure control (set up for one tomosynthesis scan to 1.5 times the dose of one mammographic view). The system reconstructed the tomosynthesis slices at 1-mm slice separation. Ultrasounds of suspicious densities or lesions were performed on Canon Aplio i600 and 400 ultrasound machines (Canon Medical Systems, Tochigi, Japan) if needed. Data were analyzed using IBM Statistical Package for Social Sciences (SPSS), Version 20.0 (IBM SPSS Statistics, Armonk, NY).

The BIRADS category was recorded separately for AV, DBT, and both. Additionally, in cases where ultrasounds were performed, the BIRADS category was also recorded. In all cases, Kappa was used to see agreement between the AV and DBT. In cases where there was a discrepancy between the DBT and AV, ultrasound was used as the gold standard to verify the accuracy of DBT versus AV.

## Results

The study included 187 subjects with a mean age of 48.8 ± 11.2 years (range: 26 to 82 years). All patients underwent AV and DBT for asymmetric densities. Five patients were excluded due to incomplete data. Among the remaining 182 patients, 84 (46.15%) were categorized as BIRADS-0, 97 patients (53.30%) as BIRADS-I, and one patient (0.55%) as BIRADS-II on additional views.

Among the asymmetric densities categorized as BIRADS-0 on AV, DBT categorized 72, six, five, and one patient as BIRADS-0, BIRADS-I, BIRADS-II, and BIRADS-IV, respectively. For densities categorized as BIRADS-1 (97) on AV, DBT categorized 10 and 87 densities as BIRADS-0 and BIRADS-I, respectively. No change among BIRADS-II and BIRADS-IV categories was noted.

The BIRADS category assigned for asymmetric densities on additional mammography views and DBT, AV alone, and DBT alone have been summarized in Table 1.

**Table 1:** Breast Imaging Reporting and Data System (BIRADS) Category Assigned for Asymmetric Densities on Additional Mammography Views and Digital Breast Tomosynthesis (DBT), Additional Views (AV) Alone, and DBT Alone

	BIRADS-0	BIRADS-I	BIRADS-II	BIRADS-IV
DBT and AV	72	6	5	1
Only AV	84	97	1	0
Only DBT	10	87		

No change in the BIRADS category was observed among BIRADS-II and BIRADS-IV. A significant difference was observed with chi-square among BIRADS categories assigned by additional views and DBT with a P-value of < .001. There was, however, a good agreement among additional views and DBT with a Kappa value of 0.767.

Eighty-nine asymmetric densities were subjected to ultrasound examination, the results of which are presented in Table 2.

**Table 2:** Cross-Tabulation Showing Frequency of Patients Who Were Assigned a Breast Imaging Reporting and Data System (BIRADS) Category on Digital Breast Tomosynthesis (DBT), Which Was Changed After Ultrasound Examination

		BIRADS on Ultrasound						Total
	BIRAD Categories	1	2	3	4	5	6	
BIRADS on DBT	0	2	44	3	21	3	1	74
	1	5	5	0	0	0	0	10
	2	0	4	0	0	0	0	4
	4	0	0	0	1	0	0	1
Total		7	53	3	22	3	1	89

A chi-square test was applied, which showed a P-value of .003, showing a significant difference between the two imaging modalities. This is mainly due to the change of BIRADS-0 after the ultrasound examination.

## Discussion

Early detection recommendations for breast cancer include awareness of early symptoms, screening by self-manual and clinical breast examination, and mammography [10]. DBT is an accessory tool for clinical use in both testing and diagnostic settings. It is a modern technique characterized by a three-dimensional (3D) reconstruction of breast tissues with high spatial resolution. This improves the visibility of breast lesions by reducing the superimposition of breast tissues, with a resultant increase in early cancer detection and reduction in false-positive findings [11]. It is composed of a rotating x-ray tube that acquires a series of low-dose projections of the compressed breast over a range of angles with either a static or a moving detector. The low-dose projection images are reconstructed with approximately 1 mm thick slices [4].

Positioning for AV can be challenging for technologists. Up to six views can be obtained in some subjects to ensure that the area in question is included. However, positioning for DBT is much simpler, being identical to positioning for routine mammography, and the problem with tissue superimposition is greatly reduced. Thus, DBT has the potential to reduce the number of additional mammographic views obtained during a diagnostic workup and improve technologist efficiency. A persistent mass or distortion detected with DBT will likely still require an ultrasound for further evaluation, which will aid in clinical assessment.

The literature has established that DBT reduces unnecessary recall due to overlapping normal tissue [12-14]. This helps in recall rates, reducing not only the cost but also the distress caused by a false-negative recall [11-12]. Unless the DBT information is clear, data from additional views may still be valuable in individual cases. The use of DBT for lesion assessment thus promises to reduce both histopathological assessments and short-term follow-up examinations, as well as patient distress and costs, while improving diagnostic accuracy.

Tomosynthesis also aids in better characterization of lesions as benign or malignant. Therefore, given our results, DBT use will reduce the number of recall examinations and lead to fewer short-interval follow-up examinations and biopsies in patients with benign pathologies [15].

Gur et al. reported higher levels of sensitivity and specificity for breast cancer detection when tomosynthesis was used compared to standard mammographic views [16]. Multiple other studies have shown that the use of DBT is equal or better than standard mammographic views for cancer detection [15-18]. Hakim et al. reported that tomosynthesis was preferred to additional views in 50% of cases compared with full-field digital mammogram and supplemented diagnostic views [19].

Provided that our results can be reproduced prospectively, and a good percentage of additional imaging can be spared, the overall radiation dose of assessment with DBT (using 1.5 times the dosage of a mammographic view) would remain more or less unchanged (compared to formerly 1.8 additional views on average) [20]. Another study has shown that by using tomosynthesis, radiologists more accurately assessed tumor measurements and tumor outlines than when using standard mammography [21]. Further dose savings appear possible by reducing stereotactic biopsies and short-term follow-up.

The radiation dose for a single tomosynthesis examination is comparable to or less than a full-field digital mammography examination [22]. According to the results of our study, we found good agreement between DBT BIRADS and AV BIRADS. Hence, DBT proved to be equivalent to, if not more equivalent to, additional imaging in the assessment of mammographically-detected asymmetric densities. The results are also comparable to earlier studies [20-21].

Our study was limited in that it was a retrospective collection of data with a small sample size. A much larger collection of cases from a wider variety of sources would strengthen our findings and improve the generalizability of our results for incorporating DBT into standard practice. The cases included in this study were from a single vendor’s equipment, and all readers were from the same institution. Our study also did not include calcified densities; hence, these results could not be implemented for calcified lesions.

## Conclusions

The study results showed that by incorporating tomosynthesis views in a standard mammographic study, we can better characterize asymmetric densities and improve BIRADS classification. This will further help reduce short-interval follow-up and recall examinations and improve breast cancer detection rates.
